# Kynurenine Metabolites and Migraine: Experimental Studies and Therapeutic Perspectives

**DOI:** 10.2174/157015911795596621

**Published:** 2011-06

**Authors:** Annamária Fejes, Árpád Párdutz, József Toldi, László Vécsei

**Affiliations:** 1 Department of Neurology, Albert Szent-Györgyi Clinical Centre, University of Szeged, Szeged, Hungary; 2 Department of Physiology, Anatomy and Neuroscience, University of Szeged, Szeged, Hungary

**Keywords:** Cortical spreading depression, glutamate, kynurenic acid, kynurenine metabolites, migraine, migraine generators, trigeminal system.

## Abstract

Migraine is one of the commonest neurological disorders. Despite intensive research, its exact pathomechanism is still not fully understood and effective therapy is not always available. One of the key molecules involved in migraine is glutamate, whose receptors are found on the first-, second- and third-order trigeminal neurones and are also present in the migraine generators, including the dorsal raphe nucleus, nucleus raphe magnus, locus coeruleus and periaqueductal grey matter. Glutamate receptors are important in cortical spreading depression, which may be the electrophysiological correlate of migraine aura.

The kynurenine metabolites, endogenous tryptophan metabolites, include kynurenic acid (KYNA), which exerts a blocking effect on ionotropic glutamate and α7-nicotinic acetylcholine receptors. Thus, KYNA and its derivatives may act as modulators at various levels of the pathomechanism of migraine. They can give rise to antinociceptive effects at the periphery, in the trigeminal nucleus caudalis, and may also act on migraine generators and cortical spreading depression. The experimental data suggest that KYNA or its derivatives might offer a novel approach to migraine therapy.

## MIGRAINE

Migraine is one of the idiopathic headache syndromes [1], and one of the commonest neurological disorders [2]. Despite intensive research, the exact pathomechanism of migraine is still not fully understood and complete preventive and attack therapy can not always be achieved. Activation of the peripheral and central arms of the trigeminal system (TS) are known to be crucial in the attack [3]. This activation may be related to cortical spreading depression (CSD) or to the activity of distinct areas of the brain stem, known as migraine generators [4, 5].

The fundamental mechanism of the migraine attack involves activation of the trigeminovascular system. Through a trigger mechanism, vasodilatation of the dural and pial blood vessels occurs, which can stimulate the perivascular trigeminal primary nerve endings. The activated nociceptors release neuropeptides at the periphery, including calcitonin gene-related peptide (CGRP), substance P and neurokinin A [6]; the levels of CGRP and substance P are elevated during migraine attacks in humans and in animal migraine models [7]. The released neuropeptides cause sterile neurogenic inflammation in the dura mater, in the course of which the blood vessels further dilate, plasma protein extravasation occurs, the mast cells degranulate and release histamine, and polymorphonuclear leukocytes appear [8]. These reactions can be observed in animal models of migraine too [9, 10]. The released inflammatory substances stimulate the trigeminal first-order neurones, leading to peripheral sensitization [11]. This usually evolves within 30 minutes, and gives rise to a throbbing head pain that is aggravated by activities that increase the intracranial pressure, including physical exercise, bending down, coughing and sneezing [12]. 

The cell bodies of the trigeminal pseudounipolar first-order neurones are located in the trigeminal ganglion (TG). The peripheral projections of these neurones partially innervate the intracranial pain structures, including the dural and pial blood vessels, the large blood vessels of the brain, the dural sinuses and the dura and pia mater, while the central projections end on the second-order neurones of the trigeminal nucleus caudalis (TNC), located in the medulla and the upper portion of the spinal cord. The activation of these first-order neurones leads to an increase in the glutamate level in the TNC [13] and, presumably *via *the N-methyl-D-asparate (NMDA) glutamate receptors [14], to activation of the second-order neurones [15]. Besides the NMDA receptors, all the other glutamate receptors are present in the TNC [16], and therefore they can also contribute to this process, which is confirmed by the fact that their antagonists are able to inhibit the increase in the number of c-Fos-immunoreactive (IR) neurones [17] and the evoked potential responses [18] in the TNC. Furthermore, the activation of second-order neurones can be modulated through α7-nicotinic acetylcholine (nACh) receptors, which act presynaptically on the transmission of nociceptive information to the central nervous system [19, 20]. 

Besides peripheral sensitization, the persistent activation of second-order trigeminal neurones evolves to central sensitization in migraineurs, with the appearance of cutaneous allodynia of the scalp and face [15, 21], when non-nociceptive stimuli produce pain. The central sensitization comprises an exaggerated sensory drive, mediated in part by glutamate receptor activation, since increases in extracellular glutamate are correlated with changes in sensory thresholds on the face of the rat [13]. Moreover, the activated second-order trigeminal neurones have functional connections to other important brain stem centres, such as the nucleus tractus solitarius, which can result in nausea and vomiting. Further activation and sensitization of the TS can provoke the sensitization of the third-order neurones from the thalamus to the cortex, which leads to other symptoms of migraine, including photophobia, phonophobia, osmophobia and allodynia of the extremities [21]. 

Migraine attacks are casually linked with the activation of distinct brain stem nuclei, known as migraine generators, which include the dorsal raphe nucleus (DRN), the nucleus raphe magnus (NRM), the locus coeruleus (LC) and the periaqueductal grey matter (PAG) [5, 22, 23], which are components of the ascending and descending pain pathways. The importance of these areas in migraine is underlined by the fact that migraine attacks could be induced in human subjects by stimulation of the PAG with an implanted electrode [24]. A possible explanation is that the above-described areas may be dysfunctional [25] and perhaps lose their natural antinociceptive function, resulting in headache. Glutamate appears at this level too since its antagonist can decrease the activity of the NRM [26], and its level is increased after stimulation of the sciatic nerve and mechanical foot shock in the LC [27] or after neuronal stimulation in the PAG [28]. 

Another potential trigger mechanism of migraine involves CSD. This is a slow continuous spread of excitation, followed by depression [29], and is accompanied by slowly-spreading cortical hypoperfusion [30]. It is widely accepted that CSD is the basis of migraine aura [31], which includes various transient neurologic symptoms, the most common of which are visual symptoms. In the process of CSD, activation of the neuronal apical dendrites [32] and astrocytes [33] seems to be important. The latter can link neuronal and vascular events [34]. Although it is not fully understood how CSD can trigger migraine attacks, under certain experimental conditions in animal models, CSD is able to activate the trigeminovascular afferents [4], increase the persistent blood flow and cause plasma protein extravasation in the dura mater [35] and hence to initiate the above-described sensitization procedures in the TS. Another connection between CSD and trigeminal activation may be glutamate and its receptors [36], which play important roles in the generation and propagation of CSD [37]. 

## ROLE OF GLUTAMATE IN MIGRAINE

Glutamate is known to play an important role in primary afferent neurotransmission and nociception [38], and numerous human and animal studies suggest that glutamate is additionally crucial in the pathomechanism of migraine [39]. Measurements of the level of glutamate in the plasma and platelets in migraine patients led to conflicting results: there have been reports of elevated basal glutamate levels in the plasma and platelets of migraineurs, which are further enhanced during the attacks [40, 41], while other studies have described lower or similar levels to those in control subjects [41, 42]. Elevated levels of glutamate in the cerebrospinal fluid have been measured during attacks in migraineurs, which favours the hypothesis of persistent neuronal hyperexcitability in the disorder [42]. The glutamate receptor antagonists can abolish the aura in patients with familial hemiplegic migraine [43] and headache [44]. Animal and human localization studies have revealed glutamate receptors in the TS [16, 45, 46, 47]. Irritation of the trigeminal nerve results in an increased glutamate level in the TNC [13]. L-Glutamate and NMDA can excite the trigeminothalamic nociceptive neurones [14, 48], and NMDA receptor activation mediates nociceptive transmission in the TNC [14]. The administration of glutamate receptor antagonists mitigated the activation of second-order neurones, i.e. the increase in the number of c-Fos-IR neurones [17, 49], the local blood flow changes [50] and the evoked potential responses [18] in the TNC and the dural plasma protein extravasation [51]. Furthermore, the NMDA receptors in the thalamus contribute to the development and maintenance of inflammation-induced hyperalgesia [52].

Glutamate and its receptors are present in the migraine generators too, and seem to be important from the aspect of nociception. For example, the broad-spectrum excitatory amino acid (EAA) antagonist kynurenic acid (KYNA) can decrease the effect of low-intensity electrical stimulation of the nucleus cuneiformis in the NRM [26], and can reduce the response of the serotoninergic neurones in the DRN [53, 54, 55, 56]. Moreover, electrical stimulation of the sciatic nerve and mechanical foot shock enhanced the rates of glutamate release from the LC [27]. The excitatory effect on the LC of glutamate released from the terminals of the nucleus paragigantocellularis, the main source of glutamate in the LC [57], was inhibited by glutamate receptor antagonists [57, 58]. Finally, in the PAG, the glutamate level was increased after neuronal stimulation [28]. These results suggest that glutamate and its receptors may well be important in the triggering of migraine attacks too, and not merely during headache. 

In the generation of CSD, a number of different ion pumps and channels are involved [59], among which NMDA receptors and therefore glutamate seem to play crucial roles: (i) NMDA receptor antagonists can inhibit CSD [60], (ii) glutamate is released during CSD under both *in vivo* and *in vitro* conditions [61, 62] and (iii) the administration of glutamate and NMDA can evoke CSD [36, 37]. One rare autosomally inherited subtype of migraine with aura is familial hemiplegic migraine. In patients with this condition, CSD may be triggered more easily presumably because the mutations involved increase the synaptic glutamate level [63]. 

Overall, it seems that glutamate is one of the key molecules in migraine at many levels of the nervous system. Its modulation may be an important means of understanding the pathomechanisms underlying the attack and it may be of potential therapeutic value in migraine.

## KYNURENINE METABOLITES

**Fig. (1):**
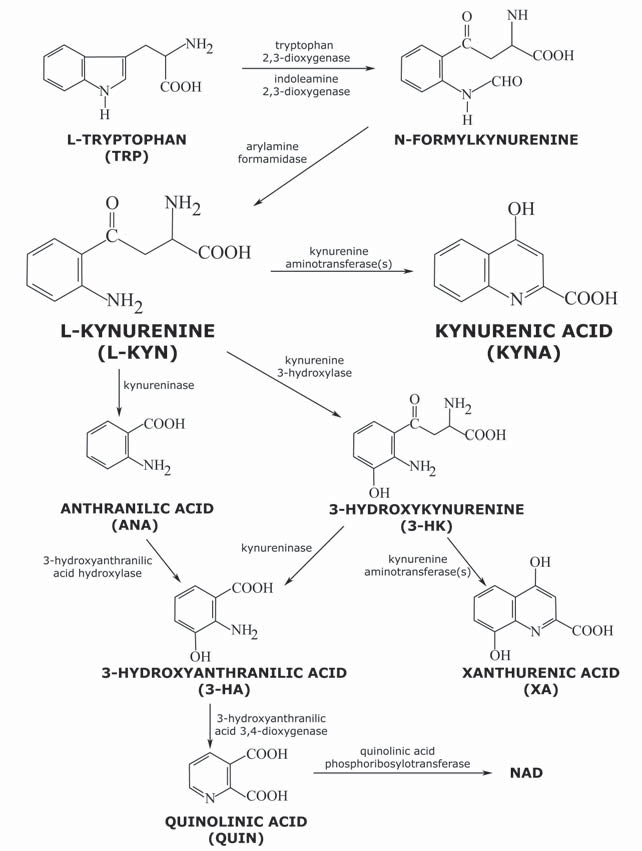
**The kynurenine pathway**.

The oxidative ring opening of tryptophan (TRP) leads to L-kynurenine (L-KYN) and the kynurenine pathway (KP) (Fig. **1**). The class of compounds known as kynurenine metabolites comprises the totality of the metabolites of the KP, the central route [64] responsible for around 95% of the TRP metabolism [65]. It takes place in the macrophages and microglial cells, and in part in the astrocytes [66, 67], and gives rise to the formation of nicotinamide adenine dinucleotide (NAD) and nicotinamide adenine dinucleotide phosphate (NADP) [68]. 

The basal compound of the KP is L-KYN, which can cross the blood–brain barrier with the aid of a neutral amino acid carrier [69]. The metabolites of the KP include 3-hydroxykynurenine (3-HK), anthranilic acid (ANA), 3-hydroxyanthranilic acid (3-HA), xanthurenic acid (XA), quinolinic acid (QUIN) and KYNA, all with neuroactive properties [70]. 

3-HK and 3-HA, generated from L-KYN, can cause neuronal damage, because they can elevate the oxidative stress level by production of free radicals [71, 72] or can provoke primary or secondary excitotoxicity [73, 74]. 3-HK is present in nanomolar concentrations in the mammalian brain, though its level can rise to the micromolar range in several pathological conditions [75]. The content of 3-HA, synthetized from 3-HK and/or ANA, likewise increases in various neurological disorders [76]. 3-HK and 3-HA have been demonstrated to cause the death of cultured neuronal cells [77, 78], the cortical and striatal neurones proving the most vulnerable to the toxic effects of 3-HK [78]. Consequently, these compounds have neurotoxic effects [74].

Transamination of 3-HK leads to XA, this generally being considered part of a detoxification process that reduces the concentration of 3-HK [79]. The role of XA in mammals is not well defined. Under physiological conditions, XA is present in the rat brain at a concentration of about 1 µM; an increase is observed in its level in the urine in an animal model of depression [80]. Administration of high doses of XA to rats seems to induce a degree of sedation and analgesia [81]. XA undergoes vesicular accumulation, is transported by neuronal cells, is present in neuronal circuits and is released *via *a calcium-dependent process in response to stimulation, these features strongly indicating a physiological role for XA in synaptic signalling [79]. 

QUIN, from which NAD and NADP are formed [68], resides in the cerebrospinal fluid in nanomolar or low micromolar concentrations [82]. When administered intrastriatally, it causes a significant destruction of neurones [73]; its excitotoxic effect is presumably exerted through agonism of the NMDA receptor [83] or stimulation of the release and inhibition of the uptake of endogenous glutamate [84]. It also induces lipid peroxidation [85, 86] and the production of reactive oxygen species [86]. Changes in the absolute or relative concentration of QUIN play an important role in certain neurodegenerative disorders [75, 87, 88]. 

In contrast with QUIN, KYNA (4-hydroxyquinoline-2-carboxylic acid) exerts a neuroprotective effect: it is able to prevent the neuronal loss in excitotoxic, ischaemia-induced and neuronal injuries [89, 90]. It is synthesized directly from L-KYN in the astrocytes and neurones [67, 91] enzymatically by the action of kynurenine aminotransferases (KATs) [92, 93], mitochondrial aspartate aminotransferase [94] and hemoperoxidases, or non-enzymatically by reactive oxygen species (ROS) [95]. Beyond this route KYNA can be produced from TRP on an additional pathway by tryptophan aminotransferase and ROS [96, 97]. Similarly to that of QUIN, the concentration of KYNA in the human brain is in the nanomolar range [98], which changes in pathological circumstances, including neurological disorders. The level of KYNA can either decrease or increase in various neurological disorders [75, 87, 99]. KYNA is one of the few known endogenous inhibitors of the EAA receptors, including the α-amino-3-hydroxy-5-methyl-4-isoxazole propionic acid (AMPA), NMDA and kainate (KA) receptor types at higher concentrations [100, 101, 102]. At around 7.9 μM it can block the NMDA receptor by attaching to its glycine-binding site [101]. As a consequence of its binding to the glutamate-binding site, KYNA may influence the receptors *via *two mechanisms: in nanomolar to micromolar concentrations, it facilitates the AMPA receptors, whereas at high concentrations, it inhibits the glutamate receptors [103]. It was demonstrated by Rozsa *et al.* [104] that KYNA in micromolar concentrations exerts a neuroinhibitory effect, while in nanomolar concentrations it behaves as a facilitator in the rat hippocampus. KYNA may therefore play an important role in the regulation (inhibition/excitation) in the neuronal network. The normal concentration of KYNA is too low to influence the EAA receptors, and the published data indicate that, even under pathological conditions, the concentration elevation will not necessarily allow KYNA to influence the co-agonist site of the NMDA receptor [105]. It has also been reported to act as a non-competitive blocker of the α7-nACh receptor [106]. This action, which may play a part in the ability of KYNA to generate a deficit in the sensory system [107, 108], has been suggested to be mediated by its binding to sites located in the N-terminal domain of the α7-nACh receptor subunit [109]. Recent results support the view that the KYNA-sensitive presynaptic α7-nACh receptors inhibit glutamate release at low concentration (30–100 nM) [105, 110]. Thus, the nACh receptors may take part in the inhibitory effects of KYNA at low concentration. KYNA could potentially have therapeutic effects in neurological disorders [75, 111, 112] *via *the above-described receptor inhibitory effects, but its use as a neuroprotective agent is rather restricted because it has only a very limited ability to cross the blood–brain barrier [69]. The experimental data suggest that peripheral treatment with L-KYN dose-dependently increases the concentration of the neuroprotective KYNA in the brain, offering an opportunity for the treatment of stroke and neurodegenerative disorders [88, 113, 114, 115]. 

Various studies have identified nACh receptors and subunits in the nociceptors of the TG at the messenger ribonucleic acid (mRNA) and protein levels [116]. The α3ß4 and α4ß2 subtypes of the nACh receptor can presumably be found on the trigeminal free nerve endings [117]. Other studies have reported that the α7-nACh receptor is likely to be present in the TG [116]. These receptors can play a role in the tonic inhibition of spinal pain, which can modulate spinal pain perception [118] and probably reduce neurogenic facial vasodilatation, presumably as a result of the decreased release of CGRP from the trigeminal afferent neurones [119]. 

## KYNURENINE METABOLITES AND MIGRAINE

### 1. Effects of Kynurenine Metabolites on First-Order Neurones

**Fig. (2):**
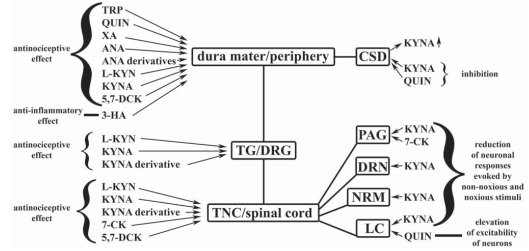
**Effects of kynurenine metabolites on the structures of nervous system, which are important in the pathomechanism of migraine and pain.** 3-HA: 3-hydroxyanthranilic acid, 5,7-DCK: 5,7-dichlorokynurenic acid, 7-CK: 7-chlorokynurenic acid, ANA: anthranilic acid, CSD: cortical spreading depression, DRG: dorsal root ganglion, DRN: dorsal raphe nucleus, KYNA: kynurenic acid, LC: locus coeruleus, L-KYN: L-kynurenine, NRM: nucleus raphe magnus, PAG: periaqueductal grey matter, QUIN: quinolinic acid, TG: trigeminal ganglion, TNC: trigeminal nucleus caudalis, TRP: tryptophan, XA: xanthurenic acid; ↑: increased concentration.

It is presumably due in part to the existence of various peripheral mechanism that TRP and some of its metabolites, including KYN, KYNA, QUIN, ANA and XA, administered intraperitoneally, can induce analgesia in both the tail-flick and the hot-plate tests, the degree and duration of analgesia varying, depending on the drug, the dose and the test [81] (Fig. **2**). The derivatives of ANA, including *N*-(3,4-dimethoxycinnamoyl)anthranilic acid (tranilast), *N*-(2,3-xylyl)anthranilic acid (CI-473, mefenamic acid) and the sodium salt of *N*-(2,6-dichloro-*m*-tolyl)anthranilic acid (sodium meclofenamate), probably act at the periphery [120], exerting both anti-inflammatory and analgesic properties, with several mechanisms of action [121, 122, 123]. 3-HA also has anti-inflammatory effects [124]. 

**Fig. (3):**
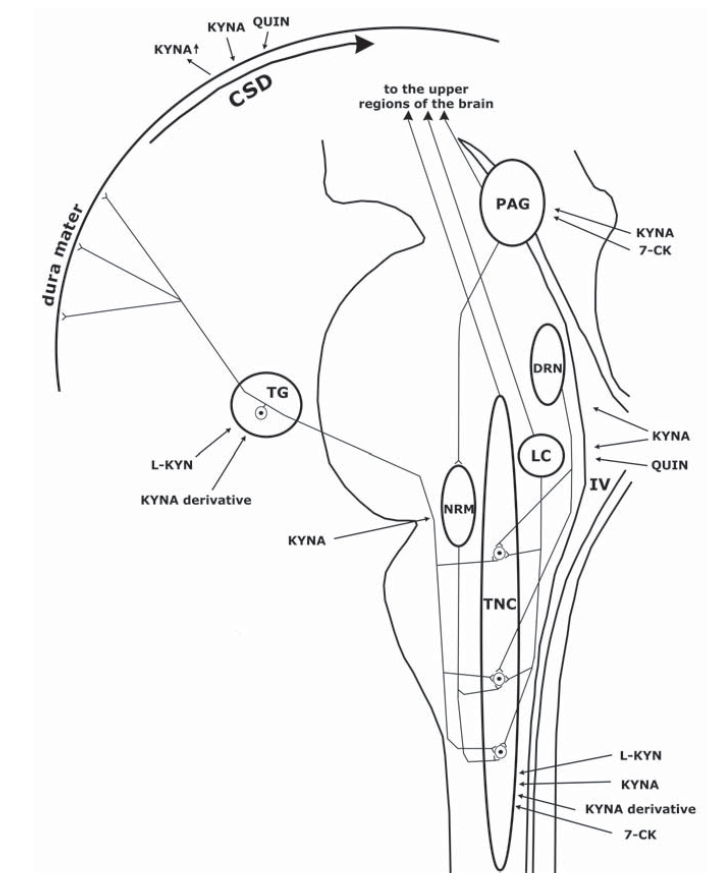
**Effects of kynurenine metabolites on the nervous structures involved in the pathogenesis of migraine.** 7-CK: 7-chlorokynurenic acid, CSD: cortical spreading depression, DRN: dorsal raphe nucleus, IV: fourth ventricle, KYNA: kynurenic acid, LC: locus coeruleus, L-KYN: L-kynurenine, NRM: nucleus raphe magnus, PAG: periaqueductal grey matter, QUIN: quinolinic acid, TG: trigeminal ganglion, TNC: trigeminal nucleus caudalis; ↑: increased concentration.

Numerous data are available in connection with the antinociceptive peripheral effect of KYNA. The intraperitoneal injection of rats with KYNA decreased the pain sensitivity in both the tail-flick and the hot-plate tests [125]. Topical intra-articularly administered KYNA, without signs of systemic side-effects, dose-dependently decreased mechanical allodynia, which manifested 30 min after the injection and the highest dose (400 μg) produced prolonged antinociception and almost total relief of allodynia [126]. A KYNA derivative, the 5,7-dichlorokynurenic acid (5,7-DCK), dose-dependently inhibited the development of the nocifensive behaviour evoked by formalin-induced tissue injury and inflammation, and reversed cold allodynia in the chronic constriction injury model, and tactile allodynia in animals subjected to spinal nerve ligation [127]. In one animal model of trigeminovascular activation after electrical stimulation of the TG, the KAT expression of the dural Schwann cells, mast cells and macrophages was decreased, presumably as a result of release from these cells; at the same time, the content of nitric oxide synthase (NOS)-IR nerve fibres in the dura mater increased, suggesting the release of nitric oxide (NO) at the periphery [128]. In another animal model of trigeminal activation, administration of the NO donor nitroglycerine (NTG), the decrease in the area covered by CGRP-IR fibres was prevented by L-KYN in combination with probenecid (PROB) and a KYNA derivative [129], the most likely explanation being that these compounds blocked the activation of first-order neurones and the consecutive release of CGRP from the nerve endings (Fig. **3**). These peripheral effects of KYNA can materialize on glutamate receptors localized at the periphery, including the dorsal root and trigeminal ganglion [130, 131], primary sensory afferents [132, 133], postganglionic sympathetic efferents [134], the temporomandibular joint [135] and Schwann cells [136] or on α7-nACh receptors located at the periphery, e.g. the trigeminal ganglion [116]. The G-protein-coupled receptor-35 (GPR35), recently identified as a receptor for KYNA [137], is expressed within nociceptive pathways, including the DRG and spinal cord, at the mRNA and protein levels [138, 139] and is negatively coupled to adenylate cyclase - cyclic adenosine monophosphate (cAMP) signalling in the DRG neurons, which can modulate nociceptive signalling [139]. KYNA proved able to inhibit the forskolin-stimulated formation of cAMP from cultured rat DRG sensory neurones *via *the GPR35 receptors and can therefore also modulate nociceptive signalling at the periphery [139]. 

### 2. Effects of Kynurenine Metabolites on Second-Order Neurones

Besides the peripheral effects of the kynurenine metabolites, several studies have confirmed that they can also act on the second-order neurones.

In behavioural examinations the intrathecal (i.t.) injection of KYNA and 7-chlorokynurenic acid (7-CK) produced dose-dependent and reversible analgesic effects in the hot-plate, tail-flick and formalin tests of nociception in mice [140] and in rats [141, 142]. Moreover, the i.t. administration of KYNA and 7-CK suppressed hyperalgesia dose-dependently in rats injected with carrageenan [125, 143], treated with i.t. strychnine [144] or after unilateral partial ligation of the sciatic nerve [145]. In mice treated i.t. with an NMDA receptor agonist, the i.t. co-administered 7-CK inhibited the nociceptive behaviour dose-dependently [146]. Finally, i.t. administration of 5,7-DCK dose-dependently reversed the hyperalgesia in hyperalgesic Mg-deficient rats [147]. However, the injection of 7-CK into the rostral anterior cingulate cortex did not affect formalin-induced acute nociceptive behaviour or electric foot shock-induced conditioned place avoidance [148] and the i.t. infusion of 5,7-DCK failed to block the glycine-induced increased pain response in neuropathic rats made by unilateral partial ligation of the sciatic nerve [149]. These results suggest that the central action of kynurenine metabolites in modulating pain perception does not extend to all brain areas that participate in nociception and is dependent on the receptors that take part in pain transmission. 

There is also evidence concerning the antinociceptive effects of kynurenine metabolites at the spinal cord level (Fig. **2**). The iontophoric administration of KYNA into the spinal cord of cats, for example, markedly reduced both the cutaneous and the muscular nociceptive responses of a wide dynamic range neurones [150] and the nociceptive responses, irregular spontaneous discharges and C-afferent-induced responses of dorsal horn neurones facilitated by the iontophoretic injection of EAA receptor agonists [151]. Further, the i.t. administration of 7-CK reduced the frequency-dependent potentiation (wind-up) to repeated C-fibre stimulation and the related post-discharges [152], but not the initial responses [153] in the nociceptive neurones located in the dorsal horn of rats. Additionally, single-unit recordings of the responses of dorsal horn neurones to C-, Aδ- and Aβ-fibre stimulation and the wind-up and post-discharge responses of the same cells facilitated by bicuculline were inhibited by 7-CK in intact anaesthetized rats [154]. KYNA pre-administered i.t. significantly reduced the total number of c-Fos-IR neurones increased by carrageenan injection into the rat paw, with a more apparent reduction in laminae I-II and IV-V [125]. *In vitro* experiments on spinal cord also suggest the antinociceptive effect of KYNA. For example, it blocked the excitation of high-threshold mechanoreceptive units by either cutaneous nerve volleys or mechanical stimulation of the skin, suppressed peripherally evoked responses to innocuous mechanical stimuli in the hamster [155] and blocked the responses to non-nociceptive and nociceptive stimulation of the skin of the leg modulated by motoneurone depolarizations and changes in extracellular potassium concentration in the frog [156]. 

The second-order nociceptive neurones of the TNC play an important role in the pathomechanism of migraine: the i.t. administration of 7-CK significantly reduced the neuronal mechanoreceptive field size and spontaneous activity increased by neonatal capsaicin treatment in adult rats [157], and intracisternally administered KYNA effectively blocked capsaicin-induced eye wipings [158] (Fig. **3**.). After systemic treatment with NTG, a well-known activator of the second-order trigeminal neurones [159], L-KYN combined with PROB attenuated the increase in the number of c-Fos-IR neurones in the TNC [160]. Similarly, at the same location, in the same experimental model, pretreatment with the L-KYN+PROB combination and a KYNA derivative, 2-(2-*N*,*N*-dimethylaminoethylamine-1-carbonyl)-1H-quinolin-4-one hydrochloride, mitigated the increase in the number of neuronal NOS- and calmodulin-dependent protein kinase II alpha-IR cells [129, 161]. Since both enzymes may play important roles in trigeminal central sensitization [162, 163], KYNA and its derivatives may exert modulatory effects on this phenomenon. KYNA alone failed to modulate c-Fos activation in the TNC in the same model [164], probably because of its poor ability to cross the blood–brain barrier, in marked contrast with its precursor L-KYN and its derivatives, which cross with ease. In another model of migraine, after electrical stimulation of the trigeminal ganglion, pretreatment with i.p. L-KYN combined with PROB mitigated the increase in the content of c-Fos-IR neurones in the rat TNC [165]. Thus, KYNA and its analogues are able to modulate second-order nociceptors in the TS. The above-described results suggest that kynurenine metabolites may have novel perspectives in the treatment of pain and migraine.

### 3. Effects of Kynurenine Metabolites on Migraine Generators

There is abundant evidence to indicate that the kynurenine metabolites are able to influence the functioning of migraine generators located at the brain stem level (Fig. **3**).

KYNA reduced the responses of serotoninergic neurones of the DRN that were evoked by phasic auditory stimuli [54], by stimulation of the lateral habenula [53], by local electrical stimulation of afferent terminals [55] and by substance P microinfusion [56]. KYNA can also abolish the activation of neurones in the NRM excited by glutamate administration [166] and by low-intensity electrical stimulation of the mesencephalic nucleus cuneiformis [26], and its injection into the PAG can modulate the excitatory and inhibitory effects of electrical and chemical stimulation of the medial preoptic nucleus of the hypothalamus on the NRM [167]. The kynurenine metabolites can modulate the LC too: for example, intracerebroventricular administration of QUIN increased the unit discharge of LC neurones [168]. However, KYNA was able to inhibit the activation of central noradrenergic neurones in the LC evoked by noxious stimulation such as electrical stimulation of the rat hindpaw [57], non-noxious and noxious cutaneous sensory stimuli [158], electrical stimulation of a rear footpad [169] and sciatic nerve stimulation [58]; noxious effect, i.e. sciatic nerve stimulation provokes activation of the catecholamine metabolism within the LC cells, which is decreased by KYNA [170]. The robust activation of the LC neurones by the direct application of KA, NMDA, AMPA or quisqualate was reduced or completely antagonized by KYNA [58, 171, 172]. KYNA was also able to inhibit the activation of the LC neurones evoked by stimulation of nucleus paragigantocellularis [57], which causes increased levels of EAAs in the LC [57, 58]. Furthermore, 7-CK prevented nociceptive behaviour (tail-flick) and pain-related changes in neuronal activity induced in the rostral ventromedial medulla by glycine or D-serine administration into the ventrolateral PAG [173]; the co-administration of KYNA with morphine in the same area enhanced the acute antinociceptive effects of morphine [174]. These results demonstrate that the kynurenine metabolites, are particularly KYNA and its derivatives, can give rise to antinociceptive effects through their influence on higher brain areas. 

### 4. Effects of Kynurenine Metabolites on CSD

There are a number of experimental data which suggest that glutamate plays an important role in the phenomenon of CSD. The glutamate level was found to be elevated during CSD [62, 61], glutamate or NMDA was able to trigger CSD [36, 37], and the NMDA, AMPA and KA receptor binding sites were increased 1 hour after the induction of CSD in rat neocortical tissues, which may be responsible for the delayed excitatory phase after it [175]. On the other hand, NMDA receptor antagonists, including the non-competitive channel blocking antagonists and competitive glutamate-recognition site antagonists, can inhibit the initiation, propagation, amplitude, frequency and susceptibility of CSD, whereas the non-NMDA receptor antagonists can not [60]. Those of the NMDA receptor antagonists that act on the NR2-B subunit may selectively inhibit the initiation and propagation of CSD [176]. These data strongly suggest that only the NMDA receptors play a role in CSD. This is further supported by the results of studies, which examined the effects of Mg^2+^ (an NMDA receptor channel blocker), and found that it can selectively inhibit glutamate-induced spreading depression (SD) [177], and that the Mg^2+^ depletion, which releases the voltage-dependent block of the NMDA receptor channel, induces CSD [178]. 

Few studies have been made of the link between CSD and the kynurenine metabolites, and the available results are conflicting (Fig. **3**). It has been established that unilateral, consecutive CSDs result in ipsilateral increases in KYNA levels in the frontal, parietal and occipital cortices [179]. Some studies have indicated that KYNA can inhibit SD under certain conditions in the turtle cerebellum [37] and in the adult rat neocortex [180], while others were not able to detect such an effect in the CA1 neurones of the rat hippocampus [181] or in neocortical brain slices [182]. Interestingly, QUIN concentration-dependently suppressed the elicitation of CSD in the cerebral cortex of the rat, presumably because of NMDA receptor desensitization [183, 184]. Since a wide range of NMDA receptor antagonists are able to inhibit electrical CSD, it is highly likely that KYNA can also do this. In experiments where KYNA was ineffective, ischaemic SD was elicited by O_2_/glucose deprivation, in which glutamate probably does not play a role, whereas it seems to be crucial in potassium-triggered SD. Consequently, KYNA and its derivatives may be of promise in the therapy against migraine aura, where important parts are played by the ion pumps and hence the ion currents. 

## CONCLUSIONS

Overall, the involvement of KP metabolites (particularly KYNA and its derivatives) at various sites of nociception and in migraine is of appreciable importance. The evidence points to the ability of these compounds to modulate migraine at several levels of the related neuronal areas, including the primary nociceptive afferents, the neurones in the TNC, and the migraine generators, and presumably at the CSD level too. KYNA and its derivatives may therefore offer new opportunities in the therapy of migraine and other diseases related to trigeminal nociception.
